# Changes in intracranial pressure gradients between the cerebral hemispheres in patients with intracerebral hematomas in one cerebral hemisphere

**DOI:** 10.1186/1471-2253-14-112

**Published:** 2014-12-03

**Authors:** Wusi Qiu, Qizhou Jiang, Guoming Xiao, Weiming Wang, Hong Shen

**Affiliations:** Department of Neurosurgery, Hangzhou Second Hospital, College of Medicine, Hangzhou Normal University, 126 Wenzhou Road, Hangzhou, 310015 China; Department of Neurosurgery, Second Affiliated Hospital, College of Medicine, Zhejiang University, 88 Jiefang Road, Hangzhou, 310009 China

**Keywords:** Intracranial-pressure monitoring, Elevated intracranial pressure, Intracranial pressure monitoring, Cerebral hemisphere

## Abstract

**Background:**

Intracranial-pressure (ICP) monitoring is useful for patients with increased ICP following hemorrhagic stroke. In this study, the changes in pressure gradients between the two cerebral hemispheres were investigated after hemorrhagic stroke of one side, and after a craniotomy.

**Methods:**

Twenty-four patients with acute cerebral hemorrhages and intracerebral hematomas who exhibited mass effect and midline shift to the contralateral side on computed tomography were selected for this study. After admission, both sides of the cranium were drilled, and optical fiber sensors were implanted to monitor the brain parenchyma pressure (BPP) in both cerebral hemispheres. All patients underwent surgical hematoma evacuations. The preoperative and postoperative BPP data from both cerebral hemispheres were collected at various time points and compared pairwise.

**Results:**

There were statistically significant differences (*P* < 0.01) in the preoperative BPP values between the two hemispheres at three different time points. Differences in the BPP values between the two hemispheres at the time of surgery, and 24 and 48 h after surgery, were not statistically significant (*P* > 0.05). The posteroperative BPPs of both hemispheres were statistically significantly lower than preoperative recordings.

**Conclusions:**

BPP sensors should be applied to the injured cerebral hemisphere, because this becomes the source of increased ICP. Hematoma evacuation surgery effectively decreases ICP and eliminates pressure gradients between the two cerebral hemispheres, consequently enabling brain shift correction.

## Background

Rapid diagnosis and management of hemorrhagic stroke is essential because it often progresses rapidly during the initial hours following onset. The application of intracranial pressure(ICP) monitoring in patients with increased ICP due to spontaneous cerebral hemorrhage is important for determining any changes in the patients’ conditions and for guiding treatment methods [1]. However, no studies to date have investigated the ICP differences between the cerebral hemispheres [2–4]. In this study, simultaneous ICP monitoring of both cerebral hemispheres was implemented in 24 patients with intracerebral hemorrhages on one side of the brain. ICP recordings were compared in order to understand pressure gradient variations between the two cerebral hemispheres.

## Clinical data and methods

### General information

This prospective study reviewed data from the three levels of first-class comprehensive hospital at Affiliated Hospital of Hangzhou Normal University and at the Second Affiliated Hospital, College of Medicine, Zhejiang University, China. According to Good Clinical Practice standards, the research protocol was approved by the Institutional Review Board and by the ethical committees of the Clinical Medical College of Hangzhou, and Declaration of Helsinki principles were strictly adhered to. Since the patients in this study were incapable of granting informed consent, the investigators obtained this from the patients’ legal guardians or by healthy proxy, before proceeding with the advised treatment.

### Patient population

Twenty-four patients who were admitted between January 2006 and January 2012 with acute cerebral hemorrhages and intracerebral hematomas were enrolled in the study.

All patients met the following criteria: supratentorial hemorrhagic stroke in the basal ganglion or brain lobe detected by computed tomography (CT) scan, with either (1) hemorrhage volume >30 ml, (2) midline shift >10 mm, or (3) obviously compressed basal cisterns.

Patients were excluded if they were either under the age of 18 years or above 65 years, or if they had any of the following: a previous disabling neurological disease, previous craniectomy, fixed dilated pupils, or a Glasgow Coma Scale score of three, with no chance of survival.

In total, 19 men and 5 women, aged between 19 and 65 years (mean ± standard deviation [5], 40.2 ± 4.5) were enrolled in the study. After onset, all patients exhibited clinical manifestations of acute intracranial hypertension including headache, nausea, vomiting, and various degrees of unconsciousness. CT examinations revealed signs of hemorrhage in one cerebral hemisphere, lateral ventricular compression on the injured side, and midline shift to the contralateral. The blood was located in the basal ganglion or in other lobes in one hemisphere, and the hemorrhage volume ranged from 30 ml to 100 ml. All bleeding was supratentorial and did not occur in the posterior fossa.

### Method of ICP monitoring

In this study, the ICP was recorded as the brain parenchyma pressure (BPP) in both cerebral hemispheres. All patients underwent surgical hematoma evacuations. Before and after surgical evacuation, nonsurgical interventions such as hyperventilation, the use of mannitol, or hypertonic saline were proposed. After admission, drilling was performed at the cranial poles on both sides in accordance with the procedures described by Gelabert [6]. Two Camino-110-4B ICP sensors were inserted into the brain tissues at both cranial poles and were connected to a Camino-MPM1 ICP monitor to record the ICP of both cerebral hemispheres simultaneously for pair-wise comparisons [7]. All patients underwent hematoma evacuation surgery within 0.5 to 45 h after the start of the ICP monitoring. Following surgery, simultaneous ICP monitoring was continued in both cerebral hemispheres for 3 d, and the data were compared pairwise.

### Statistical analysis

Monitoring data were presented as mean , and paired *t*-tests were conducted using SPSS statistical software.

## Results

### Comparison of the preoperative ICP monitoring data between the two cerebral hemispheres

In all patients, preoperative ICPs were recorded in both hemispheres at different time points. Since the timings of the craniotomies varied between patients (due to differences in severity and urgency), the number of ICP recordings for each patient was different. The first recording was performed in all 24 patients, but in 19 and 10 patients for the second and third recordings. The results of each recording are presented in Table 1: the differences in ICPs between the two cerebral hemispheres were statistically significant at all three time points (*P* < 0.01).

**Table 1:** **Comparison of the preoperative ICPs between the two cerebral hemispheres (mmHg,**  ± **SD** **)**

Side	First recording (n = 24)	Second recording (n = 19)	Third recording (n = 10)
Injured side	25.7 ± 5.7*	25.6 ± 4.8*	29.8 ± 2.7*
Contralateral side	23.5 ± 5.4	23.1 ± 4.7	27.4 ± 2.8

*P < 0.01, compared with the contralateral side.

### Comparison of the postoperative ICP monitoring data between the two cerebral hemispheres

The postoperative ICPs were recorded in both cerebral hemispheres in all patients on the day of the craniotomy and 24 and 48 h after the craniotomy. The results of each recording are presented in Table 2: the differences in the ICPs between the two cerebral hemispheres were not statistically significant at all three different time points (*P* > 0.05).

**Table 2:** **Comparison of the postoperative ICPs between the two cerebral hemispheres (mmHg,**  **± SD), n = 24**

Side	Same day of surgery	24 h after surgery	48 h after surgery
Injured side	9.6 ± 4.9*	10.2 ± 4.6*	13.5 ± 5.3*
Contralateral side	9.7 ± 4.5	10.2 ± 4.0	13.3 ± 5.4

*P > 0.05, compared with the contralateral side.

### Comparison between the preoperative and postoperative ICPs of the two cerebral hemispheres

The postoperative ICPs in both cerebral hemispheres in all patients were statistically significantly lower compared with those before surgery. Consistent with previous studies, craniotomies appeared to have significantly decreased the elevated ICP.

## Discussion

The dynamic ICP value may be more sensitive and effective in preventing secondary brain herniation in patients with hemorrhagic stroke compared with guidance directed by clinical signs and radiological indicators. Currently, there are several clinical methods commonly used for monitoring ICP [8]. Among these, intraventricular pressure (IVP) monitoring is the most reliable and accurate; therefore, IVP monitoring data are considered the gold standard for ICP [9]. However, this monitoring method has drawbacks and limitations. When ICP increases substantially, especially during brain swelling [10], the cerebral ventricles typically become very narrow under pressure, resulting in reducing success rates for ventricular punctures [11–15]. In recent years, brain parenchyma pressure (BPP) monitoring has been developed and promoted in clinical practice. Its monitoring principle is based on the fact that the brain is composed of approximately 60% water. Therefore, a specially designed optical fiber sensor can be applied to the brain parenchyma to measure hydrostatic pressure in order to determine the ICP. Brean et al. [16] simultaneously measured BPP and IVP in the same patient, and the results of the two measurements were found to be very similar. Due to the ease of BPP monitoring and because the placement of the pressure sensor is not restricted by the increased ICP, BPP monitoring is often selected over IVP monitoring. Another advantage of BPP monitoring is that a single optical fiber sensor can detect the ICP, and it simultaneously displays cerebral metabolic parameters, such as temperature, partial pressure of oxygen and carbon dioxide, and pH.

Increased ICP can be divided into two types, diffuse and local [17–21]: local ICP is more common in clinical neurosurgery. The key characteristic of local intracranial hypertension is the increased pressure originating from one intracranial chamber. This generates a pressure gradient between other chambers, often resulting in brain shift from the high-pressure chamber to the low-pressure chamber. In the past, various ICP-monitoring methods have been used to determine the mean ICP, but these methods cannot display the pressure gradients between the intracranial chambers. Transcranial Doppler ultrasound examinations performed during the swelling of one cerebral hemisphere after trauma have shown that the hemodynamics of the two cerebral hemispheres change to different degrees. This is likely to relate to the pressure difference between the two cerebral hemispheres and can be demonstrated through dynamic monitoring of BPP on both sides. The results of the current study indicated that when one cerebral hemisphere is damaged and bleeding, the injury site becomes the source of the increased ICP. The pressure gradient formed between the two hemispheres is the fundamental cause for the midline shift of the cerebral falx. Therefore, when ICP monitoring is required in patients with lesions in one cerebral hemisphere (particularly when BPP monitoring method is selected) the pressure sensor should be applied to the cerebral hemisphere with the lesion and as close to the lesion as possible. This is because the injured cerebral hemisphere is the source of the increased ICP. This is the only way to fully understand the pressure change in the local intracranial hypertension and to this method will improve the clinical relevance of ICP monitoring. Monitoring ICP is also useful when measurements are required for deciding to elect craniotomies and continued ICP measurements can help direct medical therapy post-operatively.

In this study, following craniotomies to remove the source of intracranial hypertension in patients with local intracranial hypertension, ICP monitoring revealed a significant decrease in BPP and a disappearance of the BPP difference between the cerebral hemispheres. It is generally accepted that the purpose of various surgical treatment principles for brain injuries (including hematoma evacuation, dural opening, and skull flap removal) is to lower ICP and eliminate brain compression. It has also been suggested that craniotomies can improve cerebral circulation and restore effective cerebral perfusion pressure on the lesion side. The results of the current study confirm that another therapeutic effect of craniotomies is the restoration of pressure balance between cerebral hemispheres. The consequent elimination of the pressure gradients between intracranial chambers enables correction of the brain shift. It would be better for future study to include the data of midline shift before and after surgical evacuation.

## Conclusion

In order to collect intracranial information quickly and accurately, BPP sensors should be applied to in the injured cerebral hemisphere. When injury and hemorrhage occur on one side of the brain, the injured cerebral hemisphere becomes the source of increased ICP, consequently forming a pressure gradient with the other cerebral hemisphere. Hematoma evacuation surgery effectively decreases ICP and eliminates the pressure gradients between the two cerebral hemispheres, consequently enabling brain shift correction.
